# Instagram addiction among doctors : A tunisian cross-sectional study

**DOI:** 10.1192/j.eurpsy.2023.1411

**Published:** 2023-07-19

**Authors:** S. Kolsi, N. Messedi, I. Chaari, F. Charfeddine, L. Aribi, J. Aloulou

**Affiliations:** psychiatry B department, Hedi Chaker University Hospital Center, Sfax, Tunisia

## Abstract

**Introduction:**

Instagram is one of the most popular social media platforms among young people and it has specific features leading to problematic and addictive use.

**Objectives:**

To study instagram addiction and the particularities of its use in a population of tunisian doctors.

**Methods:**

A cross-sectional, descriptive and analytical study conducted on tunisian doctors (interns, residents and university hospital doctor) during the months of septembre and octobre 2022.

An anonymous self-questionnaire with different items was used via google-forms in order to collect data that co-occur the use of instagram.

Instagram Addiction Scale (IAS) Was used to assess Instagram addiction levels. A score above 37 indicates addiction to instagram.

**Results:**

The study included 106 doctors. The average age of the first connection to instagram was 28.17 years (SD=6.93 years). The total number of friends on instagram was 240.

The average time spent on instagram was 84.5 minutes per day and the number of connections to instagram was 2.78 per day(SD=1.31).

The majority(74.5%) used the cell phone as a means of connection while being alone(73.6%).

The use of instagram was observed during the whole week(73.6%).

The mean score for addiction to instagram was 31.18(SD=11.64). Instagram addiction has been found in 42.5% and 36.8% were mildly addicted.

The instagram addiction was significantly correlated with : the age of the first connection (p=0.0001), the total number of friends on instagram (p=0.0001), time spent on that social network (p=0.0001), the number of connections to instagram per day (p= 0.0001) and the mode of connection (p=0.001).

**Conclusions:**

Our results stated that addiction to instagram affects almost half of the doctors. Many factors were contributing to this addiction.

Interventions targeting these factors seems to be crucial in order to deal with this type of addiction.

**Disclosure of Interest:**

None Declared

